# Matrix Metalloproteinases and Subclinical Atherosclerosis in Chronic Kidney Disease: A Systematic Review

**DOI:** 10.1155/2016/9498013

**Published:** 2016-03-02

**Authors:** Andreas Kousios, Panayiotis Kouis, Andrie G. Panayiotou

**Affiliations:** ^1^Department of Nephrology, Nicosia General Hospital, 2230 Nicosia, Cyprus; ^2^Cyprus International Institute for Environmental & Public Health in Association with Harvard T. H. Chan School of Public Health, Cyprus University of Technology, 3041 Limassol, Cyprus

## Abstract

*Background.* Cardiovascular disease (CVD) remains a significant problem in Chronic Kidney Disease (CKD). Subclinical atherosclerosis identified by noninvasive methods could improve CVD risk prediction in CKD but these methods are often unavailable. We therefore systematically reviewed whether circulating levels of Matrix Metalloproteinases (MMPs) and tissue inhibitors (TIMPs) are associated with subclinical atherosclerosis in CKD, as this would support their use as biomarkers or pharmacologic targets.* Methods.* All major electronic databases were systematically searched from inception until May 2015 using appropriate terms. Studies involving CKD patients with data on circulating MMPs levels and atherosclerosis were considered and subjected to quality assessment.* Results.* Overall, 16 studies were identified for qualitative synthesis and 9 studies were included in quantitative synthesis. MMP-2 and TIMP-1 were most frequently studied while most studies assessed carotid Intima-Media Thickness (cIMT) as a measure of subclinical atherosclerosis. Only MMP-2 demonstrated a consistent positive association with cIMT. Considerable variability in cIMT measurement methodology and poor plaque assessment was found.* Conclusions.* Although MMPs demonstrate great potential as biomarkers of subclinical atherosclerosis, they are understudied in CKD and not enough data existed for meta-analysis. Larger studies involving several MMPs, with more homogenized approaches in determining the atherosclerotic burden in CKD, are needed.

## 1. Introduction

Cardiovascular disease (CVD) burden is substantially higher in Chronic Kidney Disease (CKD) compared to non-CKD patients [1]. In the End-Stage Renal Disease (ESRD) population, cardiovascular mortality is the leading cause of death, and despite the recently reported improvement in survival rates, CVD in this group remains unacceptably high [2]. The increase in cardiovascular risk starts early on in CKD, with a lower estimated Glomerular Filtration Rate (eGFR) shown to be independently associated with increased cardiovascular risk [3] even at the stage of microalbuminuria [4]. CKD patients are therefore justifiably considered in the highest-risk group classification for CVD [5] and, in fact, their risk of dying from a cardiac cause actually exceeds the risk of reaching ESRD [1].

Atheromatosis and arteriosclerosis are the main underlying pathologic processes in arterial disease in CKD [6]. They are attributed to a rather complex interplay of uremia-associated risk factors that are superimposed, as the disease progresses, on the already high burden of CVD traditional factors that characterizes the CKD population [7]. Subclinical atherosclerosis, as measured by noninvasive methods such as ultrasonically determined carotid Intima-Media Thickness (cIMT), is a valid predictor of coronary heart disease and vascular events in asymptomatic individuals [8]. This is particularly important in the CKD group where the classic cardiovascular risk score approach underestimates the atherosclerotic burden [9]. Measuring subclinical atherosclerosis in CKD may significantly improve CVD risk prediction [10]. Additionally, novel early atherosclerosis biomarkers, as well as possible therapeutic targets, are greatly needed in CKD patients. Matrix Metalloproteinases (MMPs) may fall into this category of both useful markers and targets in CKD disease.

MMPs are a large family of endopeptidases that function under tight control, remodeling the extracellular matrix (ECM) and regulating the activity of many important non-ECM molecules including adhesion molecules, cytokines, and growth factors. They are classified according to their substrate specificity, sequence similarity, and domain organization into six groups: collagenases (MMP-1, MMP-8, MMP-13, and MMP-18), gelatinases (MMP-2, MMP-9), stromelysins (MMP-3, MMP-10), matrilysins (MMP-7, MMP-26), membrane-type MMPs (MMP-14, MMP-15, MMP-16, MMP-24, MMP-17, and MMP-25), and other MMPs (MMP-12, MMP-19, MMP-20, MMP-21, MMP-23, MMP-27, and MMP-28) [11]. Their proteolytic activity is regulated at transcriptional and posttranslational levels but also at the tissue level by endogenous inhibitors, known as tissue inhibitors of metalloproteinases (TIMPs 1–4) [12]. In vascular physiology and pathophysiology, they hold a prominent role by remodeling the ECM scaffold of the vessel wall and as regulators of the biological activity of nonmatrix molecules, including angiotensin-I, endothelin, TNF-*α*, and others [13–15]. Based on the emerging role of MMPs in vascular remodeling and their increased expression and activation under inflammatory and oxidative stress conditions, many studies have shown MMPs imbalance to be a key event in atherosclerosis, arterial aneurysmal formation, and plaque instability [15]. Circulating levels of various MMPs have been associated with both clinical manifestations of CVD [16, 17] and subclinical atherosclerosis [18–20] or even as predictors of outcomes following revascularization [21, 22]. Additionally, increased expression of MMPs was observed at tissue level, in human carotid, coronary, and aortic atherosclerotic lesions [23–25]. Currently, the focus is on clarifying their exact role in the disease state [26] and exploiting them in innovative diagnostic and research methodologies [27], as well as using them for prevention and therapy of vascular disease [28–30].

In CKD, a plethora of underlying factors, with preeminent toxic uremic milieu and the increased levels of proinflammatory cytokines, oxidative stress, and acidosis, maintain a state of persistent low-grade inflammation, especially in ESRD, with the addition of dialysis-related factors [31, 32]. Although this state of chronic inflammation in CKD renders MMPs attractive candidates for studies in this population and despite the mounting evidence of their role in CVD, the association between MMPs and subclinical atherosclerosis in CKD patients has not been systematically studied. To this effect, we performed a systematic literature review and evaluation of the evidence associating circulating levels of MMPs with subclinical atherosclerosis outcomes in CKD patients.

## 2. Subjects and Methods

### 2.1. Search Strategy and Selection Criteria

The electronic databases SCOPUS, PubMed, and Google Scholar were searched from inception until May 2015 using the keywords: “atherosclerosis”, “metalloproteinases”, “kidney diseases”, and “hemodialysis” either in the title or the abstract or using Medical Subject Headings (MeSH) terms. The references of eligible studies were also screened for missing articles. Inclusion criteria were CKD cohort or case-control studies involving CKD patients, reporting as one of the outcomes of interest, the relationship of circulating measurement of MMPs or their tissue inhibitors (TIMPs), and markers of atherosclerosis (i.e., IMT, plaque number, or similar atherosclerotic outcomes). The electronic search was limited to articles in the English language. The included studies were identified after two reviewers (Andreas Kousios, Panayiotis Kouis) independently screened the title and abstract of the obtained electronic search results and final selection was based on full text evaluation. A third researcher (Andrie G. Panayiotou) resolved any discrepancies.

### 2.2. Data Extraction and Quality Assessment

Two reviewers (Andreas Kousios, Panayiotis Kouis) independently extracted data regarding the studies' design, characteristics of the included CKD population, methodology for circulating MMPs levels determination, and assessment of atherosclerosis outcomes. The direction and magnitude of the association were recorded, as well as additional information such as method of statistical analysis and adjustment for potential confounders. The Newcastle-Ottawa scale for observational studies [33], which evaluates the selection of participants, the comparability of different groups, and ascertainment of exposure and outcome of interest, was utilized for the quality assessment of the included studies. In addition, a more detailed quality assessment was carried out regarding the methodology of atherosclerosis outcome evaluation based on the Mannheim Consensus criteria for carotid Intima-Media Thickness and plaque assessment [34].

## 3. Results

### 3.1. Eligible Studies

**Figure 1:**
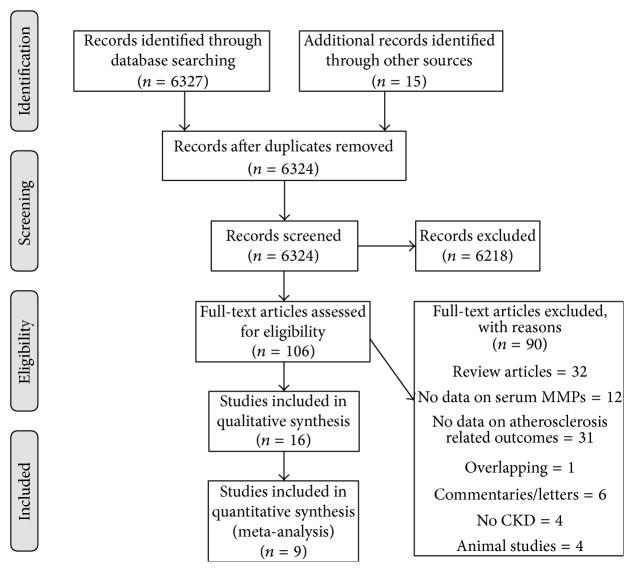
Prisma diagram for the search strategy and selected studies.

The online search retrieved 6324 items. Of them, 6218 items were excluded from further analysis based on title and abstract, while the remaining 106 were retrieved for full text assessment. Studies with overlapping populations were cross-checked and final selection was based on the number of CKD participants. Among the reports assessed in full text, 32 were literature reviews, 6 were commentaries or editorials, and another 4 were animal studies. Additionally, 12 studies did not provide data on serum concentrations of MMPs or their tissue inhibitors, 31 studies did not provide evidence on atherosclerosis related outcomes while 4 studies did not comprise a CKD population, and another one involved an overlapping population with another study. In summary, out of the total 106 reports retrieved, 16 reports were included in the qualitative synthesis and, among these, a total of 9 studies provided enough data to be included in the quantitative synthesis (Figure 1, Prisma diagram). The studies that were excluded at the last step prior to quantitative synthesis and the reason for their exclusion are presented in Supplementary Table  1 (in Supplementary Material available online at http://dx.doi.org/10.1155/2016/9498013).

### 3.2. Study Characteristics

Descriptive characteristics of the studies that were included are presented in Table 1. Four studies were carried out in Europe while the remaining studies were performed in the USA (two), Africa (two), and Asia (one). All the studies were observational and the majority of them included a CKD subgroup of participants along with age-matched healthy controls. Weber et al. evaluated the association of MMPs with atherosclerosis outcomes only in CKD stages III and IV [35] while Sánchez-Escuredo et al. evaluated MMPs and cIMT in CKD patients awaiting renal transplantation [36].

Overall, the nine studies reviewed here involved a total of 1061 participants, of whom 858 were CKD patients and 203 were healthy controls. Of the CKD patients, 450 were CKD patients already undergoing hemodialysis (HD).

The association between MMP-2 and TIMP-1 with atherosclerosis was the most frequently assessed (four studies) with MMP-9 also assessed in three. MMP-10, TIMP-2, and PAPP-A were assessed in two studies. Seven studies used cIMT as the atherosclerosis outcome and two of them also used an Atherosclerosis Score and carotid plaque number [37, 38]. The two studies included that did not measure cIMT provided data on the relationship between MMPs or their tissue inhibitors and aortic and coronary artery calcification [35] and carotid plaque presence [36]. Characteristics of studies, including atherosclerotic outcome assessed, are shown in Table 1.

MMP-2 was found to have a positive association with cIMT even after adjustment for multiple confounders in three studies [39–41] and a positive association with abdominal aortic calcification but not with coronary artery and thoracic aortic calcification [35].

The relationship of TIMP-1 with cIMT was less consistent as only one of the three studies evaluating this relationship reported a statistically significant positive association; however, it did not account for different confounders [38]. Similarly, Weber et al., who evaluated the relationship between TIMP-1 and calcification at coronary and aortic sites and included adjustment for multiple confounders, reported no statistically significant association either.

MMP-9 was found to be positively and strongly associated with cIMT, Atherosclerosis Score, and number of carotid plaques in a CKD population by Addabbo et al. [37] but this relationship was not confirmed in two additional studies evaluating MMP-9 and cIMT [39, 40]. MMP-10 was only assessed in two studies and both of them reported a positive association with cIMT in HD subgroups [38, 42] but only one of them reported a similar association in a non-HD, CKD subgroup.

Sánchez-Escuredo et al. evaluated the relationship of PAPP-A with plaque presence and reported a significant positive association in a population of HD patients awaiting kidney transplant (OR: 4.45; CI: 1.22–16.2; *P* value: 0.023) [36]. However, PAPP-A was not found to be associated with cIMT in a more recent study also involving HD patients [43].

Among the tissue inhibitors of MMPs evaluated in this review (i.e., TIMP-1 and TIMP-2), only TIMP-2 showed some evidence of a negative association with atherosclerosis as Pawlak et al. reported a negative association after adjusting for confounders between TIMP-2 and cIMT [39], although in a more recent study by the same group this finding was not repeated [40].

### 3.3. Quality Assessment

The quality assessment of the included studies was performed according to the Newcastle-Ottawa scale and the results are presented in Table 2. Overall, the included studies were characterized by good methodology and this offers some reassurance that the results presented have not been substantially influenced by bias. However, due to the substantial variability in the methodology and equipment used for the evaluation of atherosclerosis outcome, an additional table was constructed with particular emphasis on the modalities and the measurement and reporting methods used by each study (Table 3). In concordance with the Mannheim Consensus [34], most studies assessed atherosclerosis in longitudinal view on the far wall and common carotid artery (CCA) was the most commonly used anatomical site followed by carotid bulb (CB) and the internal carotid artery (ICA). However, few studies reported whether measurements were obtained at the end of diastole or whether measurement was obtained in a blinded fashion.

## 4. Discussion

This systematic review evaluated the published evidence on the association between circulating levels of MMPs and subclinical atherosclerosis in CKD patients. We identified only nine observational studies that adequately addressed this relationship. Furthermore, the vast majority of studies were also characterized by a small sample size as most of them included less than 100 CKD patients. cIMT was the main measure of subclinical atherosclerosis reported and MMP-2 and TIMP-1 were the most commonly assessed metalloproteinases.

Although the number of studies providing the same data on MMP-2 was too small for a formal meta-analysis, the overall consistent direction and magnitude of the association of MMP-2 with cIMT reported in the different studies suggest that this is positively associated with subclinical atherosclerosis in CKD patients. It is however important to note that two out of the four studies reporting on MMP-2 were in hemodialysis patients only. On the contrary, most of the studies that evaluated TIMP-1 and subclinical atherosclerosis did not find any significant relationship, while for the remaining MMPs, the low number of studies identified does not allow for any inferences regarding their association with subclinical atherosclerosis.

Studies involving CKD patients that did not use atherosclerosis measures as an outcome were excluded at the last step, prior to quantitative synthesis, in order to limit the results of this study to objective atherosclerosis measures as opposed to clinical or self-reported measures such as “history of CVD.” Notably, in these studies, circulating levels of MMP-2 were associated with previous history of CVD in a non-HD CKD population [44] and in a Peritoneal Dialysis (PD) population [45], providing further supporting evidence for MMP-2 association with CVD in CKD (Supplementary Table  1). For consistency, we also excluded studies that had measured MMP expression in vessel tissue instead of circulating concentrations. Although tissue expression level is a direct evidence of MMP implication in the pathophysiology of atherosclerosis, it is not easily transferrable in the clinical setting as a biomarker. Furthermore, studies that involved pediatric CKD patients instead of adults were also excluded. Interestingly, only one study was found to report an association between serum measurements of MMPs and atherosclerosis markers in pediatric CKD patients, making a separate review of these findings not possible [46]. Overall, although this approach limits the number of informative studies reviewed here, it allowed us to answer the more precise question on the association between circulating MMPs and subclinical atherosclerosis in adult CKD patients.

Regulation of MMPs expression and activity in physiological or pathological vascular remodeling is induced by hemodynamics, injury, inflammation, and oxidative stress [15, 47, 48]. In CKD, a condition where these processes are enhanced, it is expected that MMP dysregulation is intensified, particularly in late CKD stages and HD. Persistent, low-grade inflammation in CKD is attributed to the production of proinflammatory cytokines combined with their decreased renal clearance, the CKD-associated metabolic acidosis, the uremic milieu induced oxidative and carbonyl stress, the chronic or frequent recurrent infections, and thrombotic events [32]. In addition, dialysis-related factors, such as membrane biocompatibility, water and dialysate purity, and microbiological quality, further contribute and sustain inflammation in ESRD [31]. This uremia-inflammation interplay in CKD underlies the accelerated atherosclerosis and increased IMT, the arterial stiffening, and increased vascular calcification of both intima and media and impairs the vascular repair process with the detrimental consequences of neointimal hyperplasia [49]. Moreover, plaque morphology, composition, and vulnerability differ in CKD, as coronary and carotid plaques of CKD patients were shown to be more calcified, more unstable, and frequently ruptured and containing less fibrous tissue [50–52]. Central to the pathogenesis of these processes and plaque formation are the endothelial cell (EC) dysfunction and vascular smooth muscle cell (VSMC) migration and their phenotypic shift to a more proliferative and secretory state [49, 53].

Activated MMPs participate in both early and late stages in atherosclerosis progression. Their cleaving of ECM and non-ECM molecules induces the pathogenic phenotypic shift of ECs and VSMCs and facilitates increased endothelial inflammation and permeability, intimal-medial thickening, fibrosis, calcification, and stiffening [26, 54]. MMPs 1, 2, 8, 9, and 12 are mostly implicated in these processes with MMP-2 and MMP-9 having a prominent role [26]. In later stages of atherosclerosis, MMPs contribute to reducing the atherosclerotic plaques' fibrous cap, [55] thus rendering plaques more unstable and prone to rupture [56]. In CKD patients, only few studies have examined the levels of circulating MMPs compared to controls demonstrating increased circulating MMP levels in CKD, particularly those of MMP-2, MMP-9, and MMP-10 [57, 58]. Additionally, MMP-2 and MMP-9 were shown to be upregulated focally in uremic vessels in two studies by Chung et al. [59, 60]. MMP-2 was upregulated in arteries of ESRD patients and activated MMP-2 was strongly correlated with arterial stiffness in dialyzed patients [60] (Supplementary Table  1). MMP-2 and MMP-9 were upregulated in diabetic CKD arteries and correlated with stiffening and endothelial dysfunction [59] (Supplementary Table  1).

As research is ongoing on the development of cardiovascular risk markers in CKD patients [7], MMPs stand to serve as potential biomarkers for atherosclerosis and cardiovascular risk assessment in this high risk group. In order for a potential biomarker to be approved for clinical use, it needs to be confirmed through rigorous testing of multiple subjects and testing should be characterized by reproducibility, good sensitivity, and specificity [61]. The limited number of studies identified in this review reflects the fact that the level of evidence is still quite low for use of MMPs as biomarkers for atherosclerosis in CKD patients, although the accessibility and relatively low cost of circulating MMPs measurements along with knowledge of the disease mechanisms argue about the benefit of additional and larger studies involving CKD patients. Moreover, such studies would provide further insight into their contribution to the higher CVD burden in CKD and, more importantly, would pave the way for their use in therapeutic interventions [30] or even their targeted and specific inhibition [62].

Although the majority of the studies reviewed here are characterized by good overall methodology according to the Newcastle-Ottawa scale criteria, we have identified additional parameters relating to the performance of atherosclerosis assessment that vary between studies and may introduce additional variability in the estimated relationship between circulating MMPs and subclinical atherosclerosis. As most of the studies used cIMT and plaque measurements as surrogates for subclinical atherosclerosis, it is important to highlight the necessity of a homogenized approach for image acquisition, data analysis, and reporting methods, as well as the use of unified criteria to distinguish early atherosclerotic plaques from increased IMT [34]. With regards to IMT measurement, the Manheim Carotid Intima-Media Thickness and Plaque Consensus report proposes the site of measurement to be the far wall of the CCA. Mean IMT values across the CCA may be less susceptible to errors compared to maximum values and composite measures of IMT and plaque should be avoided. Plaque assessment should include the location, thickness and area, and plaque number and should be scanned in longitudinal and cross sections [34]. In most of the reviewed studies, although IMT was measured in the far wall of CCA, there was considerable variability in methodology and poor plaque assessment. Furthermore, circulating levels of MMPs are influenced by environmental, genetic, disease, and drug related factors and although evaluating each of these factors individually is beyond the scope of this review; they need to be carefully examined in future study designs involving CKD populations [63]. Additionally, variations in sample collection methodology and preanalytical care have been found to significantly affect MMPs levels with serum samples reported to have higher mean values compared to plasma samples [64, 65]. The majority of the included studies in this review had measured MMPs levels in serum [36, 38–43], with only two studies using plasma [35, 37]. Although we cannot exclude the possibility of such discrepancies in explaining part of the heterogeneity in the results, it seems unlikely that they would explain all of it as similar heterogeneity exists in the results obtained from studies that used serum. Also, variations in MMPs levels could arise from the status of recruited patients as it is suggested that hemodialysis may affect MMP levels, especially MMP-2, MMP-9, and their inhibitors [66, 67]. Additionally, all studies included patients with a history of CVD. However, only six out of the nine studies reported the prevalence of CVD history in their patients groups which ranged between ~8% and 80%, while the cross-sectional design of the studies further limits the causal inferences that could be made. Finally, none of the studies performed* a priori* power analysis in order to estimate the appropriate sample size and the possibility of publication bias cannot be excluded as almost no study included in this review reported only a negative association between MMPs levels and subclinical atherosclerosis.

Despite the extensive study of MMPs and their role in the atherosclerotic process in both animal models [68] and human studies, there are disproportionately fewer published studies of the atherogenic effects of MMPs in patients with CKD. This is in keeping with a well described phenomenon of underrepresentation of CKD patients in cardiovascular disease studies [69] despite the growing global burden of kidney disease [70] and the high prevalence of CKD among CVD patients [71]. Nonetheless, based on their central role in arterial wall remodeling, MMPs demonstrate great potential for further studies in CKD, a condition where the main drivers for MMP dysregulation, such as inflammation and oxidative stress, are intensified. Their linkage to early atherosclerotic change, reflected in established but often not easily accessible subclinical atherosclerosis markers, provides the basis for MMPs use as biomarkers or even as pharmacological targets of cardiovascular disease in CKD patients.

To this effect, we have systematically reviewed the literature and critically appraised all studies addressing the association of various MMPs with subclinical atherosclerosis in CKD patients. We aimed to help structure the knowledge derived from human studies in the field and identify potential candidate MMPs for further research. Moreover, several methodological caveats were identified with regard to IMT measurement and sampling. Future research initiatives in this field are thus urgently needed and would benefit by addressing the methodological issues identified in this review, during the study design process. Overall, these findings are highly relevant in view of the undiminished interest in MMPs and the need for novel approaches to address the significant problem of CVD in Chronic Kidney Disease.

## 5. Conclusions

In summary, the published evidence reviewed here demonstrates that circulating MMPs levels could potentially be of use as biomarkers of subclinical atherosclerosis in adult CKD populations. MMP-2 shows the greatest promise although most of the other MMPs or their tissue inhibitors are mostly understudied in the CKD population and no inferences about their potential can be made. Studies characterized by larger and well defined CKD populations and involving several MMPs and a consistent and homogenized assessment of different measures of subclinical atherosclerosis such as IMT and plaque burden are urgently needed.

## Supplementary Material

Supplementary Table 1 summarizes the basic characteristics of studies excluded at the last step prior the quantitative synthesis along with the primary reason for their exclusion.

## Figures and Tables

**Table 1:** Characteristics of included studies.

Number	Author, country (Year)	Participants (*n*)	Age	Evaluated MMPs	Outcome		Association in CKD/HD patients	*P*	Direction
					IMT	Plaque/other			
1	Pawlak et al. [39] Poland (2004)	Total: 58 HD: 38 Controls: 20	HD: 59 ± 15 C: —	MMP-2 MMP-9 TIMP-1 TIMP-2	✓	—	*cIMT* MMP-2: *β* = 0.596 (0.278) (adjusted) MMP-9: *r* : −0.135 (unadjusted) TIMP-1: *β* = −0.312 (0.238) (adjusted) TIMP-2: *β* = −0.767 (0.276) (adjusted)	0.04 NR 0.20 0.010	↑ *↔* *↔* ↓

2	Addabbo et al. [37] USA (2007)	Total: 108 CKD: 75 Controls: 33	CKD: 52 ± 16	MMP-9	✓	✓	*cIMT* MMP-9: *r* = 0.30 (unadjusted) *Plaque number* MMP-9: *r* = 0.30 (unadjusted) *Atherosclerosis Score* MMP-9: *r* = 0.37 (unadjusted)	0.009 0.009 0.001	↑ ↑ ↑

3	Pawlak et al. [40] Poland (2008)	Total: 62 HD: 42 Controls: 20	HD: 59 ± 18 C: 53 ± 15	MMP-2 MMP-9 TIMP-2 TIMP-1	✓	—	*cIMT in HD* MMP-2: 0.402 (0.143) (adjusted) MMP-9: NR TIMP-1: 0.097 (−0.175) (adjusted) TIMP-2: *r* = 0.248 (unadjusted)	0.012 — 0.59 NR	↑ — *↔* *↔*

4	Nagano et al. [41] Japan (2009)	Total: 129 CKD: 99 Controls: 30	CKD: 58.3 ± 17.9 C: 56 ± 5.5	MMP-2	✓	—	*cIMT* MMP-2: *β* = 0.240 (adjusted)	0.04	↑

5	Coll et al. [38] Spain (2010)	Total: 378 HD: 217 CKD I–III: 43 CKD IV-V: 68 Controls: 50	HD: 64.7 ± 12 CKD I–III: 59.6 ± 11 CKD IV-V: 69.2 ± 12 C: 64.9 ± 3	MMP-8 MMP-10 TIMP-1	✓	✓	*cIMT in HD* MMP-8: NR (unadjusted) MMP-10: *r* = 0.16 (unadjusted) TIMP-1: NR (unadjusted) *cIMT in CKD* MMP-8: NR (unadjusted) MMP-10: NR (unadjusted) TIMP-1: *r* = 0.32 (unadjusted) *Atherosclerosis Score* MMP-8: 1.15 (0.77–1.73) MMP-10: 1.57 (1.06–2.32) TIMP-1: 1.00 (0.65–1.54)	NR 0.01 NR NR NR 0.03 0.02 0.47 0.97	*↔* ↑ *↔* — — ↑ ↑ *↔* *↔*

6	Sánchez-Escuredo et al. [36] Spain (2010)	Total: 93 RT: 93	RT: 54 ± 12	PAPP-A	—	✓	*Plaque presence* PAPP-A: OR: 4.45 (1.22–16.2) (adjusted)	0.02	↑

7	Belal et al. [42] Egypt (2014)	Total: 60 CKD: 20 HD: 20 Controls: 20	CKD: 49 ± 6.6 HD: 52 ± 6.7 C: 49 ± 6.5	MMP-10	✓	—	*cIMT in CKD* MMP-10: *r* = 0.697 (unadjusted) *cIMT in HD* MMP-10: *r* = 0.836 (unadjusted)	<0.001 <0.001	↑ ↑

8	Weber et al. [35] USA (2014)	Total: 103 CKD III: 56 CKD IV: 47	CKD III: 66.1 (12) CKD IV: 68.0 (9.3)	MMP-2 TIMP-1	—	✓	*Abdominal aortic calcification* MMP-2: *β* = NR (adjusted) TIMP-1: *β* = NR (adjusted) *Coronary artery calcification* MMP-2: *β* = NR (adjusted) TIMP-1: *β* = NR (adjusted) *Thoracic aortic calcification* MMP-2: *β* = NR (adjusted) TIMP-1: *β* = NR (adjusted)	0.02 0.06 0.90 0.27 0.18 0.16	↑ *↔* *↔* *↔* *↔* *↔*

9	Issac et al. [43] Egypt (2014)	Total: 70 HD: 40 Control: 30	HD: 45 (30.5–55.8) C: 36.5 (28.8–46.8)	PAPP-A	✓	—	*cIMT* PAPP-A: No association	NR	*↔*

CKD: Chronic Kidney Disease, HD: hemodialysis, C: controls, MMP: Metalloproteinases, IMT: Intima-Media Thickness, NR: not reported, RT: renal transplant.

**Table 2:** Quality assessment of the included studies (Newcastle-Ottawa scale).

Number	Author	Year	Newcastle-Ottawa scale scores			
			Selection	Comparability	Exposure	Summary
1	Pawlak et al. [39]	2004	4	2	2	8
2	Addabbo et al. [37]	2007	4	2	2	8
3	Pawlak et al. [40]	2008	4	2	2	8
4	Nagano et al. [41]	2009	3	2	3	8
5	Coll et al. [9]	2010	4	2	2	8
6	Sánchez-Escuredo et al. [36]	2010	2	2	2	6
7	Belal et al. [42]	2014	3	1	2	6
8	Weber et al. [35]	2014	2	2	3	7
9	Isaac et al. [43]	2014	3	1	2	6

Selection criteria (4): adequate case definition, representativeness of cases, selection of controls, and definition of controls. Comparability criteria (2): control for factor A and an additional factor B on the basis of the design or analysis. Exposure criteria (3): ascertainment of exposure, the same method for cases and controls, and nonresponse rate.

**Table 3:** Subclinical atherosclerosis assessment of the included studies based on the Manheim Consensus.

Number	Author (Year)	Tools	Angle	Anatomical site	Walls used	IMT and plaque assessment		Measurements	Quantitative measures of plaques	Measurement during end diastole	Single observer/blinded
						cIMT or IMTmax	Plaques assessed separately				
1	Pawlak et al. [39] (2004)	NR	L	CCA (B)	FW	NR	No	Mean of 2 measurements per site	No	NR	NR NR

2	Addabbo et al. [37] (2007)	NR	NR	CCA (B) CB (B) ICA (B)	FW	cIMT	Yes	Mean of 6 measurements per site DCCA	Number of plaques	Yes	Single/blinded

3	Pawlak et al. [40] (2008)	NR	L	CCA (B)	FW	cIMT	No	Mean of 2 measurements per site	No	NR	Single/blinded

4	Nagano et al. [41] (2009)	NR	L	CCA	LW MW	NR	NR	Mean of 6 measurements of CCA	No	Yes	Single/blinded

5	Coll et al. [38] (2010)	SA	L	CCA (B) CB (B) ICA (B)	FW	cIMT	Yes	NR	No	Manheim Consensus	NR Blinded

6	Sánchez-Escuredo et al. [36] (2010)	NR	NR	CCA (B) CB (B) ICA (B)	FW	cIMT	If IMT > 1,2 mm	Mean of 6 measurements per site	No	Yes	Single/NR

7	Belal et al. [42] (2014)	A	L	CCA (B)	FW	cIMT	Yes	Maximum of 2 measurements per site	No	NR	NR NR

8	Weber et al. [35] (2014)	—	—	—	—	—	—	—	—	—	—

9	Issac et al. [43] (2014)	NR	L, T	NR	NR	NR	No	IMT (B), CSA (B)	No	NR	NR NR

NR: not reported.

A: automated, SA: semiautomated.

L: longitudinal, T: transverse, and CS: cross-sectional.

CCA: common carotid artery, CB: carotid bulb, ICA: internal carotid artery, and B: bilateral.

FW: far wall, NW: near wall, LW: lateral wall, and MW: medial wall.

CSA: cross-sectional area, DCCA: internal diameter of the common carotid artery.
