# Supplementary material for: Matrix Metalloproteinases and Subclinical Atherosclerosis in Chronic Kidney Disease: A Systematic Review
Source: Int J Nephrol. 2016 Mar 2;2016:9498013. doi: 10.1155/2016/9498013 (PMC4793143; doi:10.1155/2016/9498013)
Supplement: Supplementary file 1 — Supplementary Table 1 summarizes the basic characteristics of studies excluded at the last step prior the quantitative synthesis along with the primary reason for their exclusion. [file 9498013.f1.pdf]

Supplementary Table 1: Studies excluded from quantitative synthesis and reason for their exclusion

#	Study	Participants	Age	MMPs	Outcome	Association in CKD/HD patients (magnitude)	P value	Direction	Details for exclusion
1	Fatema K.,(45)<br>Japan<br>(2004)	Total: 70<br>CKD: 30<br>HTN: 40	CKD: 62 ± 12<br>HTN: 60 ± 10	MMP-1<br>TIMP-1<br>TIMP-1/MMP-1	CV-IBS	**CV-IBS in CKD (after HD)**<br>MMP-1: NR<br>TIMP-1: NR<br>TIMP-1/MMP-1: r= - 0.52	-<br>-<br><0.05	-<br>-<br>↓	Non-atherosclerosis specific outcome
2	Pawlak K.,(46)<br>Poland<br>(2005)	Total: 68<br>HD: 50<br>Controls: 18	HD: 58.0 ± 13<br>Controls: 54 ± 7	MMP-9<br>TIMP-1<br>TIMP-1/MMP-9	History of CVD	**History of CVD in HD**<br>MMP-9: NR (unadjusted)<br>TIMP-1: NR (unadjusted)<br>TIMP-1/MMP-9 ratio: nr (unadjusted)	>0.05<br>>0.05<br>>0.05	↔<br>↔<br>↔	Non – atherosclerosis specific outcome.
3	Peireskova M.,(47)<br>Czech Republic<br>(2009)	Total: 124<br>CKD: 80<br>Controls: 44	CKD: 52±16<br>Controls: 58±10	MMP-2<br>PAPP-A	History of CVD	**History of CVD in CKD**<br>MMP-2: NR (unadjusted)<br>PAPP-A: NR (unadjusted)	<0.001<br><0.001	↑<br>↑	Non – atherosclerosis specific outcome.
4	Chung A.,(38)<br>Canada<br>(2009)	Total: 24<br>Diabetic CKD: 8<br>Non-Diabetic CKD: 8<br>Controls: 8	Diabetic CKD: 55 ± 8<br>Non-Diabetic CKD: 57 ± 11<br>Controls: 50 ± 8	MMP-2<br>MMP-9	Arterial stiffness<br>Arterial calcification	**Arterial stiffness (PWV) in CKD**<br>MMP-2+: MMP-9: r = 0.683 (unadjusted)<br>**Arterial Calcification(Phosphate) in CKD**<br>MMP-2+: MMP-9: r = 0.513 (unadjusted)	0.005<br>0.04	↑<br>↑	MMPs expression levels, not serum levels
5	Chung A.,(37)<br>Canada<br>(2009)	Total: 70<br>CKD: 17<br>HD: 23<br>Controls: 30	CKD:54 ± 2.6<br>HD: 49 ± 2.8<br>Controls:30	MMP-2	Arterial stiffness<br>Calcium deposit	**Arterial stiffness in CKD**<br>MMP-2: r = 0.326<br>**Arterial stiffness in HD**<br>MMP-2: r = 0.384<br>**Arterial stiffness in PD**<br>MMP-2: r = 0.796	0.22<br>0.19<br>0.006	↔<br>↔<br>↑	Not serum level of MMP-S, Expression levels in arteries
6	Pawlak K., (48)<br>Poland<br>(2010)	Total: 88<br>PD: 70<br>Controls: 18	HD: 50.89<br>Controls: 47.94 ± 6.60	MMP-2<br>MMP-9<br>TIMP-1<br>TIMP-2	History of CVD	**History of CVD in PD**<br>MMP-2: β = 0.365 (adjusted)<br>MMP-9: NR<br>TIMP-1: NR<br>TIMP-2: β = 0.292 (adjusted)	0.02<br>-<br>-<br>0.03	↑<br>-<br>-<br>↑	Non – atherosclerosis specific outcome.
7	Sun Y.,(49)<br>China<br>(2012)	Total: 34<br>HD: 15<br>Controls: 19	HD: 62.6 ± 10.3<br>Controls: 60.1 ± 10.5	MMP-2<br>MMP-9<br>TIMP-2<br>TIMP-3<br>MMP-2/TIMP-2	cIMT<br>Atherosclerosis Score	**cIMT**<br>MMP-2/TIMP-2: OR: 16.43 (2.02-18.37)<br>**Coronary Atherosclerosis Score**<br>MMP-2/TIMP-2: OR: 5.57 (1.32-7.59)	<0.01<br>0.01	↑<br>↑	Not serum level of MMP-S, Expression levels in veins

CKD: Chronic Kidney Disease, HD: Hemodialysis,PD: Peritoneal Dialysis, HTN: Hypertension, CVD: Cardiovascular Disease, MMP: Metalloproteinases, cIMT: carotid Intima Media Thickness, CV-IBS: cardiac cycle-dependent variation of ultrasonic integrated backscatter, r: Correlation, OR: Odds Ratio, β: Regression Coefficient, nr: non-reported
